# Digital twin key technology on rare earth process

**DOI:** 10.1038/s41598-022-19090-y

**Published:** 2022-08-30

**Authors:** Hui Yang, Zhiqin Kuang, Jianyong Zhu, Fangping Xu, Feiping Nie, Shuchen Sun

**Affiliations:** 1 School of Electrical and Electronic Engineering, East China Jiaotong University, Nanchang, 330013 China; 2 Key Labtotary of Advanced Control and Optimization of Jiangxi Province, Nanchang, 330013 China; 3 School of Compute, Northwestern Polytechnical University, Xi’an, 710072 China; 4 School of Materials & Metallurgy, Northeastern University, Shenyang, 110004 China

**Keywords:** Electrical and electronic engineering, Chemical engineering

## Abstract

Digital twin can be defined as a digital equivalent of an object of which it can mirror its behavior and status or virtual replicas of real physical entities in Cyberspace. To an extent, it also can simulate and predict the states of equipment or systems through smart algorithms and massive data. Hence, the digital twin is emerging used in intelligent manufacturing Systems in real-time and predicting system failure and also has introduced into a variety of traditional industries such as construction, Agriculture. Rare earth production is a typical process industry, and its Extraction Process enjoys the top priority in the industry. However, the extraction process is usually characterized by nonlinear behavior, large time delays, and strong coupling of various process variables. In case of failures happened in the process, the whole line would be shut down. Therefore, the digital twin is introduced into the design of process simulation to promote the efficiency and intelligent level of the Extraction Process. This paper proposes the techniques to build the rare earth digital twin such as soft measurement of component content, component content process simulation, control optimization strategy, and virtual workshop, etc. At the end, the validity of the model is verified, and a case study is conducted to verify the feasibility of the whole Digital twin framework.

## Introduction

Currently, rare studies deal with the simulation and prediction of the complete manufacturing process. Even little study has been done on process optimization based on process simulation data. Rare Earths (RE), sometimes known as industrial vitamins, are widely applied in a variety of areas, including metallurgy, high-speed trains, and the national defense military industry. The Cascade Extraction Process, which is a cascade of several extraction tanks, is frequently used to increase the purity of rare earth in Chinese RE facilities. Furthermore, the Rare Earth Extraction Process (REEP), as a typical complex industrial process, is characterized by nonlinear behavior, long-time delays, and significant coupling of numerous process variables. In this study, we propose a DT system for REEP that incorporates inspection, virtual inspection, control, and process simulation modules. Cascade extraction is separated into thousands of extraction sub-processes, which is the principal reason of the lengthy process and over-reliance on human operation for failure judgment relating to the factory’s normal operation.

With the advancement of information technology, such as the industrial internet, the internet of things, and artificial intelligence, the concept of smart manufacturing is increasingly becoming such a reality. Germany, a powerful manufacturing country, suggested the ’industry 4.0’ concept^1^. One of the key challenges for smart manufacturing is to connect the physical and virtual spaces. It is necessary to set up Cyber-physical systems^2^, even Cyber-physical production systems^3^. Currently, The importance of digital twin (DT) is increasingly recognized by both academia and industry. many DT applications have been successfully implemented in equipment maintenance, production health management, process simulation, auxiliary design, fault diagnostics, and so on^4^.

The DT may be traced back to a product life-cycle management program lectured by Dr. Michael Grieves,who proposed an unique notion called “Information Mirroring Model”,which was then refined to “Digital Twin”^5^. Yang covered essential DT technologies such as data capture, transmission, and processing, data-driven and model fusion collaborative control, virtual interaction and collaboration, and its applications in smart manufacturing, product development, and smart cities, among others^6^.

This paper proposes four key methods to build a rare earth digital twin system to achieve the functions of real-time sensing, rapid prediction, control optimization and efficient inspection of the digital twin system: a soft measurement method of solution component based on the color characteristics of rare earth solution is proposed to obtain the solution component content in the rare earth extraction process in real time, a component content prediction method based on mechanism compensation is proposed to simulate the component content change of the extraction solution In order to improve the control effect, a control optimization method based on case search is proposed, and a virtual plant using virtual reality technology is proposed to facilitate quick understanding of the working conditions and equipment status.

The paper’s overall structure is as follows: A literature review on digital twin shop intelligent production lines technology and Digital twin technology for the process industry are provided in “Related works” section “rare earth production DT framework” section describes the architecture of a rare earth digital twin system. “Key technologies” section introduces and discusses four key enabling technologies, including soft measurement of rare earth component content, as well as the modeling approach of process simulation and the principle of parameter optimization “Model validation and case study” section presents illustrative applications and discussions to verify the proposed model. Finally, conclusions are presented in “Conclusions” section.

## Related works

Previously, More applications for digital twins can be found in the fields of equipment maintenance and flaw diagnosis. The Airframe Digital Twin (ADT), which is envisioned to be an ultra-realistic, cradle-to-grave computer model of an aircraft structure, was built by Gockel to analyze the aircraft’s capacity to satisfy mission criteria^7^. Bielefeldt then ingeniously constructed a DT of an aircraft wing subjected to flight loading and replicates the behavior of these localized particles while simultaneously lowering computing time^8^. Wang introduced a data-driven DT of the rotor system for accurate diagnosis and adaptive deterioration analysis of rotating equipment since this rotor system emulates an imbalance fault and its advancement, unbalanced quantification^9^. Jiang presented a single description and standardization of DT, which is used in a 110 kV substation’s designed prognosis and health management (PHM) system^10^. Li employed the notion of a dynamic Bayesian network to construct an adaptable probabilistic model for diagnosis and prognosis, and it showed the Feasible technique using an aircraft wing fatigue crack growth example^11^.

With advancements in virtualization, sensor technologies, and computing capacity, the notion of DT has grown to design, create, and operate complex systems by providing a secure virtual area for testing and validation^12^. Virtual reality (VR) technology captures human movements in a virtual environment, which are subsequently replicated by a real robot^13^. Bilderberg and Malik discuss an object-oriented and event-driven simulation of a flexible assembly cell coordinated with a robot to execute assembly duties alongside humans^14^. Corral-Acero created a visual DT for cardiology diagnosis and uses computer models and artificial intelligence to properly forecast cardiology. They give remedies that are personalized to each patient and optimize the healthcare system’s efficacy and efficiency^15^. Kang used DT technology to create a multimedia knowledge-based bridge health monitoring system that evaluates aberrant bridge circumstances and suggests the best time for maintenance^16^.

Scholars mainly work on the application of the digital twin in the Manufacturing industry. The implement of DT in manufacturing can optimize production processes and reduce energy consumption and costs . Tao conducted a framework to construct a digital twin system that included a real workshop, a virtual workshop, a database, and a service system^17^. Stark focused on the theoretical underpinning for the DT design framework and offered an 8-dimension Digital Twin model. The DT design components and their effect factors on the DT 8-dimension model were used to the smart manufacturing cell’s development and ramp-up operations^18^. Jones summarized important vocabulary, and associated procedures, and lists 13 major features such as physical Entity, virtual Entity, and so on^19^. The use of simulation techniques brings digital twins to life and allows them to be tested. Schluse pioneered the notion of EDTs(experimental digital twins) as a new structuring element for simulation-based systems engineering processes and their interdisciplinary and cross-domain simulation, which enables comprehensive simulations on the system level^20^. A DT case for a welding manufacturing line is created using an application framework and a virtual model^21^. Wu introduced four important technologies for achieving real-time monitoring based on DT, including data modeling and transmission, event-driven virtual and real mapping, workshop logic modeling, and information visualization^22^. Considering the influence of carbon emissions from manufacturing processes, Zhao presented a method for dynamically optimizing machining process parameters to decrease carbon emissions based on real-time observation of the machining circumstances^23^. Zhang presented a special framework for simulation optimization utilizing a virtual workshop in response to several issues encountered during the design stage of a dual-manipulator cooperative unit^24^.

The current state of digital twin technology is mostly employed in the discrete industry. Due to the complexities of process modeling, the process industry, is still missing in DT trials. Among the six essential technologies advocated by Li for process industry DT are data intelligent perception, multisource heterogeneous data integration, data-efficient transmission, digital twin creation, enhanced interaction, and transformation application^25^. Zhou streamlined the raw materials delivery schedule at an ironmaking factory with five sintering machines and seven blast furnaces utilizing cloud computing and DT technology to cut production expenses to reduce the mean coke ratio of an ironmaking plant^26^. Soares showed how a digital twin was successfully implemented to simulate a four-stage multi-effect evaporation train from an industrial sugar-cane processing facility^27^. There is presently no reference model especially established for risk control and prevention in the oil and gas industries. Bevilacqua created a Digital Twin reference model to offer conceptual criteria for DT deployment for risk prediction and prevention^28^.

## Rare earth production DT framework

**Figure 1:**
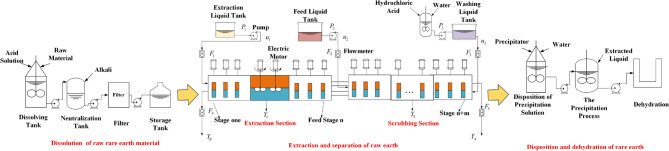
The Main procedure of rare earth production.

Figure 1 depicts the REEP (rare earth extraction process), which includes a dissolve circuit, extraction circuit, disposal circuit, and dehydration circuit. In an acid solution, the feed liquid and raw material powder are dissolved. In the dissolution process, the raw material powder is mixed with acid and water, and then neutralized with alkali to a certain pH value, and then precipitated and filtered to obtain the next stage of the raw material. In the extraction process, the solution configured in the previous stage is separated from a variety of separation products through the action of multi-stage extraction tank. These separated products can be obtained by adding precipitating agent in the precipitation process and stirring to obtain various desired products.

**Figure 2:**
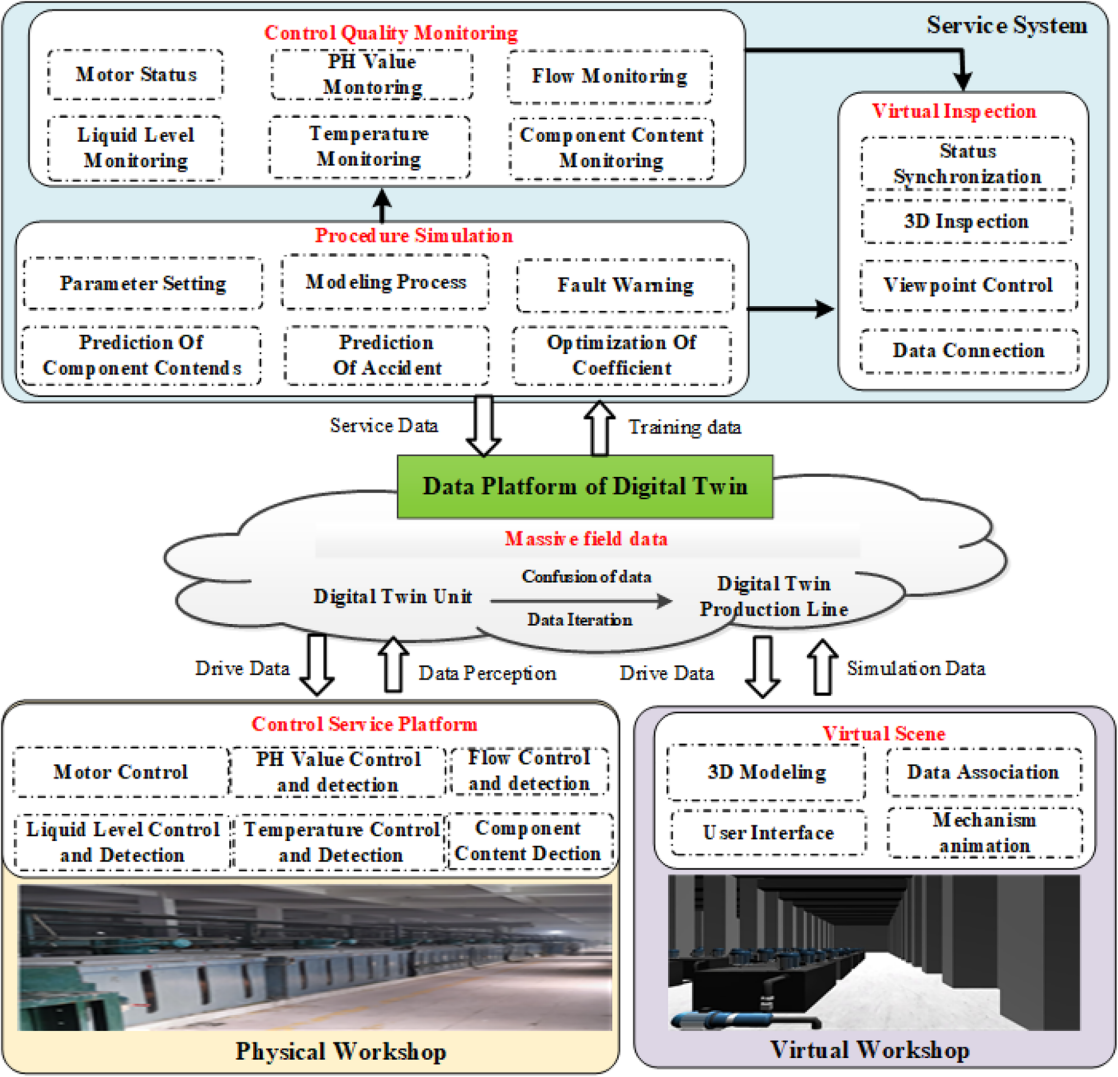
Basic framework of rare earth DT.

Figure 2 depicts the four key components of the DT architecture, which comprise a physical workshop, a virtual workshop in digital space, DT service systems, and dynamic data bases. Firstly, the physical workshop transmits the process index control data such as motor, PH, temperature, level, temperature and the collected component content data to the database for easy use by the service system and the virtual system. Then the virtual equipment in the virtual shop will synchronize the collected data in the database and realize the overrun warning and the corresponding process animation.

Finally, according to the real-time data, the service system can optimize the control index to facilitate the setting of control parameters and simulate the component content of the extraction process at all levels, while the user can quickly inspect the virtual workshop through the virtual inspection module. Taking the rare earth extraction process as an example, the digital twin system data flow is described as follows.

(1) The physical workshop realizes the control of motor, dosing pump and solenoid valve, and gets the real-time data such as solution component content of detection level and extraction tank level, temperature, inlet and outlet flow through the soft measurement system of solution component content, which is transmitted to the digital twin data platform. (2) The digital twin data platform will update the collected data into the database of the virtual workshop and the database of the service system. (3) The virtual workshop synchronizes the process simulation data from the data platform to the corresponding extraction tank with real-time data. (4) The service system includes control optimization, virtual inspection module, and process simulation module. The process simulation module can read real-time data from the DT data platform and then simulate the component content of each extraction level based on the algorithm. The control optimization module will calculate the optimal control strategy based on the real-time control data and the case-based control optimization strategy.

**Figure 3:**
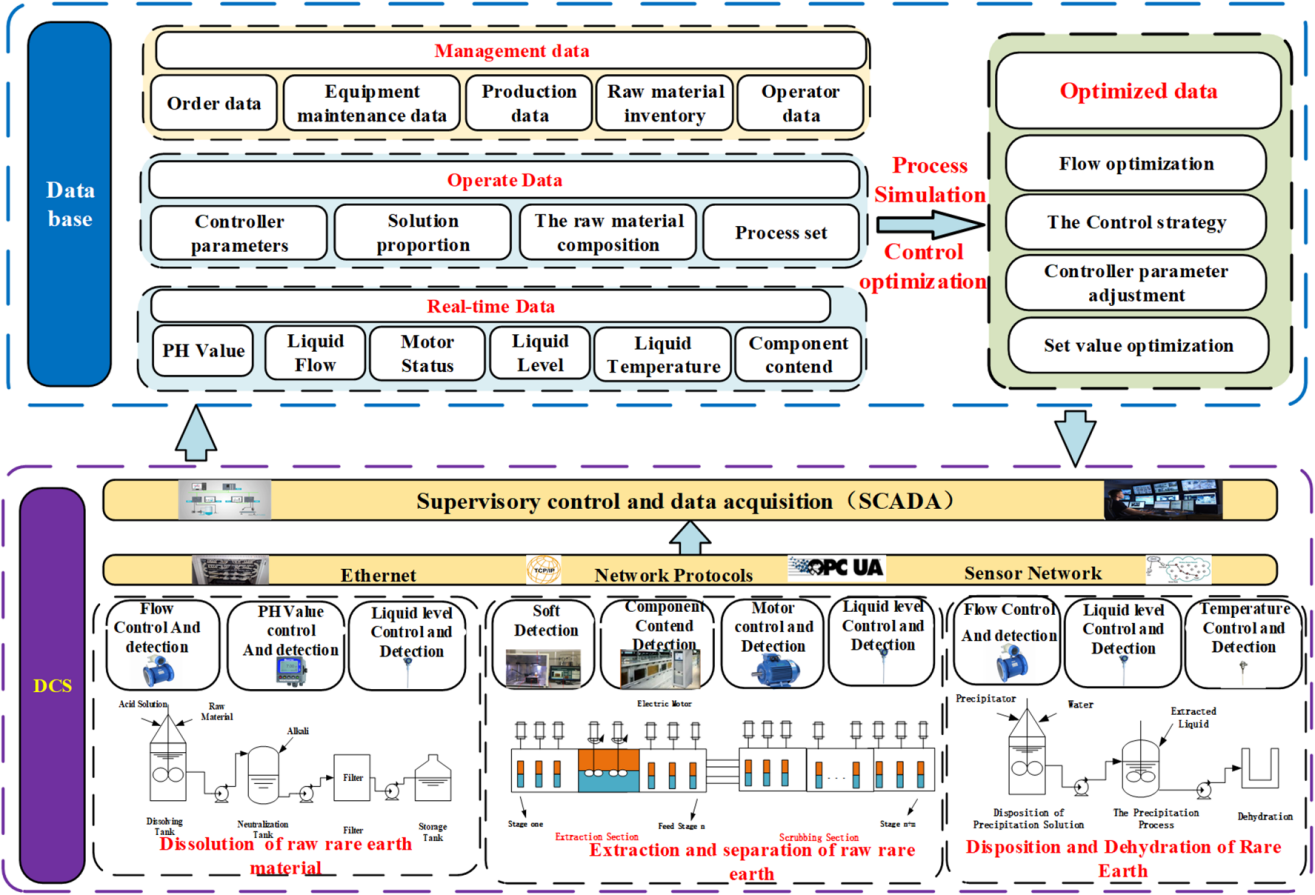
Framework for real-time collection, composition, management data of workshop.

Figure 3 presents the Framework for real-time collection, composition, management data of workshop. The framework is divided into two parts: DCS and database, where the DCS is responsible for flow, level, temperature, motor control and process index detection in each production step. The control and inspection data are transferred to the SCADA system^29^ via Ethernet and sensor networks and then uploaded to the database. The database contains real-time data, management data, operation data, and optimization data. The real-time data mainly includes the actual site control and testing data, and the operation information includes the solution proportioning, controller parameters, raw material component content and other artificially set data. Management data includes order, inventory, operator, equipment, and raw material data. Optimization data is used to store the parameter settings of the control optimization strategy such as controller settings, flow rate settings, and to send them to the SCADA system to guide the production.

## Key technologies

### Soft measurement of rare earth component content

In actual production, process engineers mainly use the color change of the extracted rare earth solution to determine the component content and abnormal working conditions. Inspired by this, Yang proposed a soft measurement model of component content using the color characteristics of the extracted solution^30^. Zhu further improved the accuracy of the soft measurement model by considering the interference generated by light changes on the solution image acquisition^31^. The soft measurement method proposed in this paper can effectively decrease the influence of illumination on the detection, and the detection accuracy is better.

The soft measurement method is used to detect the color features of the liquid to be tested and then estimate component amount. However, detection errors will arise in the field inspection due to the fading of the light source and the hostile environment. To solve this issue, the initial picture must be compensated before modeling. it is ought to set an objective function based on the Grey Edge algorithm, establish the parameters to be improved, and then apply the Grey Edge algorithm based on the Genetic Algorithm (GA) to optimize the illumination compensation model. To use Extraction of RGB features and HSI traits from light-compensated acquisition photos as the soft measurement model’s input variables. The component content is gently measured using the weighted least squares support vector machine (WLSSVM). To guarantee that the recovered color features are accurate, the lighting conditions at the time of data collecting must be consistent, hence color constancy is required for color feature extraction. The color constancy computation is a two-step method that begins with estimating the picture’s light color and ends with using the diagonal model to adjust the image to the standard light. The Grey Edge technique is a well-known unsupervised lighting estimating algorithm. The color constancy approach derived from the gray world hypothesis and the gray edge method is as Eq. (1).

1 $$\begin{aligned} \left( \int \left| \frac{\partial ^{n}f^{\sigma }(X)}{\partial X^{n}}\right| ^{p}dX\right) =ke^{n,p,\sigma } \end{aligned}$$

$f^{\sigma }=f\bigotimes G^{\sigma }$ show the convolution of the image f and the Gauss filter $G^{\sigma }$ and $\partial ^{n}(.)/\partial X^{n}$ refers the n order derivation. The color under two diverse Illumination conditions can be translated by a diagonal matrix. For an instance, the color of the image*f*under the light $e^{u}=[R^{u},G^{u},B^{u}]$ can convert to the color $f^{c}$ under the light $e^{c}=[R^{c},G^{c},B^{c}]$. only needs to be multiplied by a diagonal matrix, which is as Eq. (2).

2 $$\begin{aligned} f^{c}=\begin{bmatrix} R^{c}/R^{u}&{}0 &{}0 \\ 0&{} G^{c}/G^{u}&{}0 \\ 0&{} 0&{} B^{c}/B^{u} \\ \end{bmatrix} \end{aligned}$$

According to the production requirements of the actual site, only the middle solution image is valid for the acquired image, so we only need to calculate the error for the area of the solution region. The color of the central region of the image to be estimated is $(R_{a},G_{a},B_{a})$, the average RGB value of the image region after the algorithm is $(R_{c},R_{c},R_{a})$ Angle error is calculated by Eq. (3):

3 $$\begin{aligned} E_{a}=cos^{-1}\left( \frac{(R_{a},G_{a},B_{a})\bullet (R_{c},G_{c},B_{c})}{\sqrt{R_{a}^{2}+G_{a}^{2}+B_{a}^{2}}\times \sqrt{R_{c}^{2}+G_{c}^{2}+B_{c}^{2}}}\right) \times \frac{180^{\circ }}{\pi } \end{aligned}$$

Pre-correction color $f^{u}=[R^{u},G^{u},B^{u}]$ is known, only estimate the unknown light source in the image to be corrected $e^{u}=[R^{u},G^{u},B^{u}]$and standard light sources in standard images $e^{c}=[R^{c},G^{c},B^{c}]$,The corrected color can be obtained $f^{c}=(R_{c},G_{c},B_{c})$ Thus, the image correction problem based on illumination estimation is transformed into an optimization problem containing three parameters with the following optimization formulas as Eqs. (4)–(6).

4 $$\begin{aligned} min E_{a}= & {} cos^{-1}\left( \frac{(R_{a},G_{a},B_{a})\bullet (R_{c},G_{c},B_{c})}{\sqrt{R_{a}^{2}+G_{a}^{2}+B_{a}^{2}}\times \sqrt{R_{a}^{2}+G_{a}^{2}+B_{a}^{2}}}\right) \times \frac{180^{\circ }}{\pi } \end{aligned}$$

5 $$\begin{aligned} s.t.\begin{pmatrix} R_{c} \\ G_{c} \\ B_{c} \end{pmatrix}= & {} \begin{bmatrix} R^{c}/R^{u}&{}0 &{}0 \\ 0&{} G^{c}/G^{u}&{}0 \\ 0&{} 0&{} B^{c}/B^{u} \\ \end{bmatrix}\begin{pmatrix} R_{u} \\ G_{u} \\ B_{u} \end{pmatrix} \end{aligned}$$

6 $$\begin{aligned} ke^{n,p,\sigma }= & {} \left( \int \left| \frac{\partial ^{n}f^{\sigma }(X)}{\partial X^{n}}\right| ^{p}dX\right) ^{1/p} \end{aligned}$$

These three parameters $n,p,\sigma$ can be used as a set of independent variables, and the best estimate of the actual light color can be obtained by parameter optimization through genetic algorithms(GA), particle swarm optimization (PSO), differential evolution and other optimization algorithms.According to the optimal combination $n,p,\sigma$. The estimated light color can be obtained using above equation. After substituting the above results into the transformation matrix The final corrected color is obtained $f_{c}^{'}=(R^{'}_{c},G^{'}_{c},B^{'}_{c})$. 

The H, S and I color characteristic components are extracted from the images of rare earth solutions acquired at industrial sites under the HSI color space. Since each value of HSI has different degrees of influence on the component content, each color component is weighted. Similarly, RGB features can be added and the relationship between the solution image color and component content can be expressed as Eq. (7).

7 $$\begin{aligned} y=f(w_{h}C_h,w_{s}C_s,w_{i}C_i,w_{R}C_R,w_{G}C_G,w_{B}C_B) \end{aligned}$$

$w_h,w_s,w_i,w_R,w_G,w_B$ denote the weights of the H, S and I components of the HSI color space, respectively, $C_h,C_s,C_i,C_R,C_G,C_B$ The H, S and I components of the HSI color space and R G B components of the RGB color space are represented respectively. soft measurements of rare earth component content are mainly established, with a nonlinear relationship between the solution color HSI characteristics and the rare earth component content. The nonlinear relationship can be modeled using nonlinear regression, neural network methods.Due to the limitations of the field process, it is difficult to collect a large amount of continuous data as the training set of the neural network, so the model of soft measurement uses WLSSVM. this method features fast learning capability, simple calculation and is suitable for small sample objects.

### Process simulation of the extraction process

**Figure 4:**
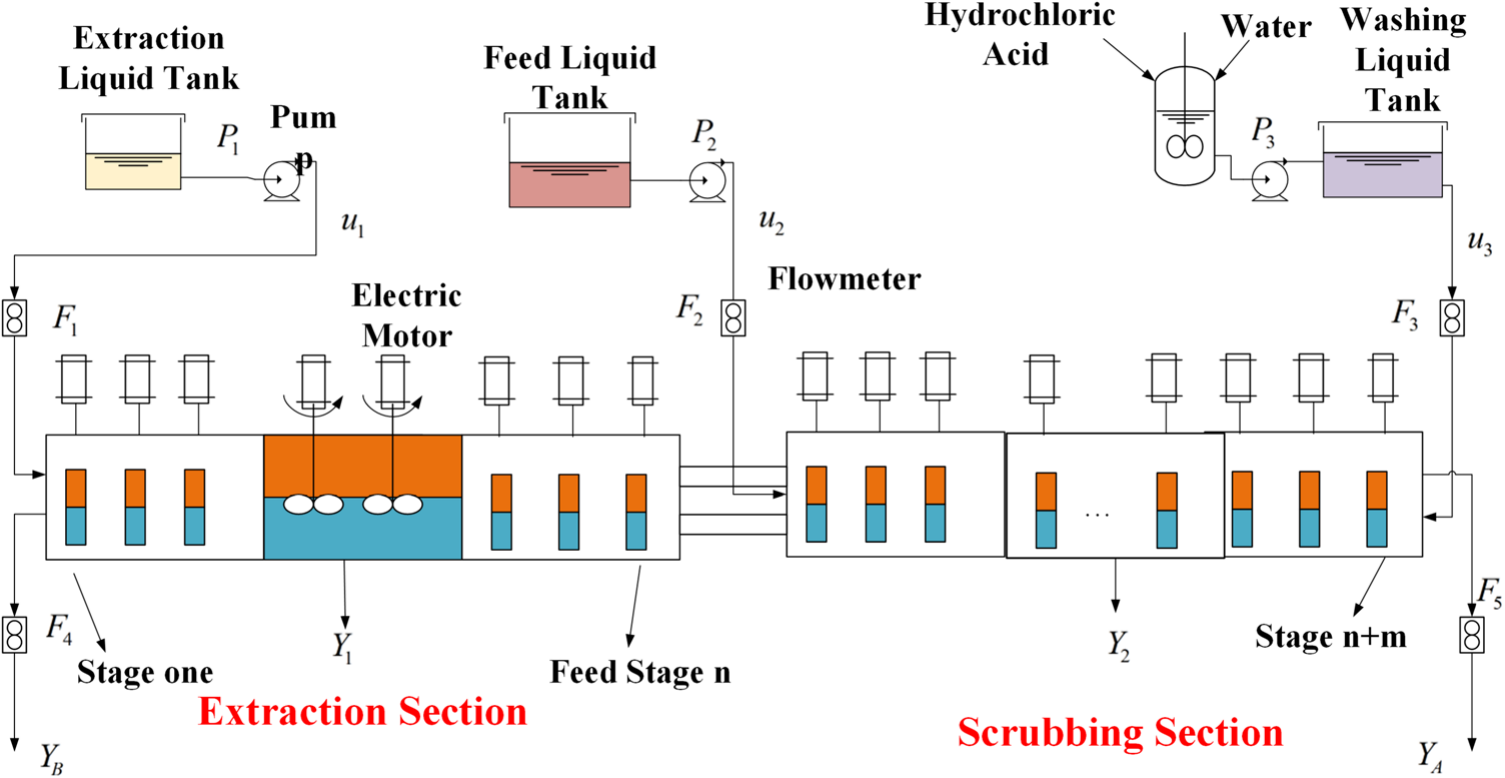
Structure of rare earth extraction process.

Rare earth, a vital raw material, has been widely employed in the production of military projects, electronic and communication equipment. The process of rare earth production can be classified a series of sub-processes. What matters most is Extraction Process which characterized by nonlinear behavior, large time delays, and strong coupling of various process variables. The extraction process has the features of long process flow, complex mechanism and many influence factors. Figure 4 shows the flow chart of rare earth extraction process. The process consists of an extraction stage and a washing stage. The mixed liquid of raw material liquid is poured into meddle stage, the extraction solvent P507 and washing liquid HCL are separately added into first stage and last stage. hard extracted product $$Y_B $$ in aqueous phase can be exported ,and the easy extracted product $$Y_A$$ in organic phase is originated from the last stage. The organic phase flows in the opposite direction of the aqueous phase.

An accurate Mathematical model is critical in anticipating the states of component content in each mixer-setter in order to simulate the component content. To simulate the extraction process, Wu developed a primary model that relies on mass and element balance^32^. Most academics deny the accuracy, yet it sheds insight on a suitable strategy for rare earth extraction simulation. JIA developed a soft-sensor model technique by incorporating the subtraction clustering algorithm^33^. Yang proposed a model that was based on an enhanced principal model with optimized parameters^34^. The previous process simulation method mainly uses a static mechanism method, which cannot adjust the model according to the actual field data, resulting in large errors in the predicted component content.In this paper, the approaches are utilized to develop a dynamically optimized model of process simulation which could further improve the accuracy of process simulation Training with historical data Compared to previous studies.

When the extraction system reached stability, the organic phase component was set to Y and the aqueous phase component to X; A denotes easy extracted component content, B denotes hard extracted component content, N denotes the number of components, I denotes the number of components, and F denotes the raw material composition. There are n extraction steps and m scrubbing stages. Since its material liquid remains invariant after extraction, the following formulas for the separation coefficient of various components may be obtained as Eqs. (8)–(10).

8 $$\begin{aligned} \beta _{(i+1)/i} =\frac{Y_{i+1}\times X_{i}}{Y_{i}\times X_{i+1}} \end{aligned}$$

N refers the last component, i refers component i.

9 $$\begin{aligned} \beta _{(1)/i}= & {} \frac{Y_{i}\times X_{1}}{Y_{1}\times X_{i}} \end{aligned}$$

10 $$\begin{aligned} \beta _{(i)/N}= & {} \frac{Y_{N}\times X_{i}}{Y_{i}\times X_{N}} \end{aligned}$$

To describe the relationship of single component between aqueous phase and organic phase, there are some formulas about the transformation with separation factor as Eqs. (11)–(12).

11 $$\begin{aligned} Y_{i}= & {} \left( X_{i}\times \prod _{i=1}^{N}\beta _{1/i}\right) /\sum _{i=1}^{N}\left( X_{i}\times \prod _{i=1}^{N}\beta _{1/i}\right) \end{aligned}$$

12 $$\begin{aligned} X_{i}= & {} \left( Y_{i}\times \prod _{i=1}^{N}\beta _{1/N}\right) /\sum _{i=1}^{N}\left( Y_{i}\times \prod _{i=1}^{N}\beta _{1/N}\right) \end{aligned}$$

According to the insufficient extraction, a compensation coefficient K have to be added in extraction coefficient to improve accuracy of model. the expression like Eqs. (13)–(14).

13 $$\begin{aligned} Y_{i}= & {} \left( X_{i}\times \prod _{i=1}^{N}K*\beta _{1/i}\right) /\sum _{i=1}^{N}\left( X_{i}\times \prod _{i=1}^{N}\beta _{1/i}\right) \end{aligned}$$

14 $$\begin{aligned} X_{i}= & {} \left( Y_{i}\times \prod _{i=1}^{N}K*\beta _{1/N}\right) /\sum _{i=1}^{N}\left( Y_{i}\times \prod _{i=1}^{N}\beta _{1/N}\right) \end{aligned}$$

To increase the accuracy of this model, an improved PSO (Particle Swarm Optimization) was employed in the optimization of compensation coefficient. It is necessary to set fitness function of optimization like following depiction. The fitness function of extraction stage as Eqs. (15)–(16).

15 $$\begin{aligned} minJ= & {} \sum _{i=1}^{L}\left( Y_{i}-\left( X_{i}\times \prod _{i=1}^{N}K*\beta _{1/i}/\sum _{i=1}^{N}\left( X_{i}\times \prod _{i=1}^{N}K*\beta _{1/i}\right) \right) \right) ^{2} \end{aligned}$$

16 $$\begin{aligned} minG= & {} \sum _{i=1}^{N-L}\left( X_{i}-\left( Y_{i}\times \prod _{i=1}^{N}K*\beta _{1/N}/\sum _{i=1}^{N}\left( Y_{i}\times \prod _{i=1}^{N}K*\beta _{1/N}\right) \right) \right) ^{2} \end{aligned}$$

An appropriate compensation coefficient requires the constant iteration through the optimization algorithm and massive date. Here presents the main process: In the initial stage of building mathematical model, the principal model with compensation coefficient was built before Application, the coefficient will be iterated by update parameters.

Compared the common PSO model, improved PSO model based on functional inertia weight and constant constriction factor to optimize compensation coefficient which is more excellent in effect of optimization. This paper is enlightened by the asynchronously improved PSO model applied in text feature selection and utilize it in compensation coefficient optimization^35^.

Common PSO model creates a large number of particles, which move in whole solution space with a fixed law. Each particle own a couple of messages about their position and fitness which also influence other particles to adjust their position and velocity of moving. The velocity of particle i expresses as $V_{i}=(V_{i1},V_{i2},\ldots ,V_{iD})$, the loction of particle*i* express as$(X_{i1},X_{i2},\ldots ,X_{iD})$, the optimal location of particle i expresses as $P_{i}=(p_{i1},p_{i2},\ldots ,p_{iD})$ it is also regarded as $p_{best}$The best optimum location is $P_{g}=(p_{g1},p_{g2},\ldots ,p_{gD})$ or express as $g_{best}$. Each particle own a single fitness calculated from fitness function. This kind of Improved PSO concentrate on the promotion of update formulae of the dimension*d* in Eqs. (17) and (18):

17 $$\begin{aligned} v_{id}= & {} w\times v_{id}+c_{1}\times Rand()\times (P_{id}-x_{id})+c_{2}\times Rand()\times (P_{gd}-x_{id}) \end{aligned}$$

18 $$\begin{aligned} x_{id}= & {} x_{id}+v_{id} \end{aligned}$$

In standard PSO, *Q* refers particle quantity,*w* refers inertia weight $c_{1}$ and $c_{2}$ both refers acceleration constant, $v_{max}$ is the maximum velocity, $G_{max}$ refers the maximum number of iterations *rand*() or *Rand*() both refers the random functions with values in [0,1].

Due to massive data conducted in production process, common PSO model fail to rapidly search the global optimum through the fixed generation. the constriction factor *K* is conduced into Eq. (19) show the improved velocity formula.

19 $$\begin{aligned} v_{id}=K[v_{id}+c_{1}\times Rand()\times (P_{id}-x_{id})+c_{2}\times Rand()\times (P_{gd}-x_{id})] \end{aligned}$$

In the early generation, the particle needs to move in a wide range to search the optimum location.it needs to develop the precision within a small range to determine the best single point. Hence, the constriction factor keep a large value in early iteration, and adjust its values with the generation change. the functional constriction factor is presented as Eq. (20). refers the iterations.

20 $$\begin{aligned} K=\frac{cos(\Pi /G_{max})\times T+2.5}{4} \end{aligned}$$

To Improve asynchronously inertia weight and constriction factor, the principal that original speed influenced by inertia weight, the convergence of PSO is affected by constriction can to be described as Eq. (21).

21 $$\begin{aligned} \left\{ \begin{matrix}v_{id}=w\times v_{id}+c_{1}\times Rannd()\times (P_{id}-x_{id})+c_{2}\times Rand()\times (P_{gd}-x_{id}) if T<\frac{G_{max}}{2}\\ v_{id}=K[\alpha \times v_{id}+c_{1}\times Rannd()\times (P_{id}-x_{id})+c_{2}\times Rand()\times (P_{gd}-x_{id})] if T\ge \frac{G_{max}}{2} \end{matrix}\right. \end{aligned}$$

$\alpha$ is the default original velocity of particle. The change way of appears in Eq. (22).

22 $$\begin{aligned} \left\{ \begin{matrix}w=w_{end}+(w_{start}-w_{end})(1-(T/G_{max})) ifP_{gd}\ne x_{id} \\ w=w_{end} if P_{gd}=x_{id}\end{matrix}\right. \end{aligned}$$

$w_{end}$ and $w_{start}$ both are set by researchers, which determine the range of inertia weight. To predict the future behavior of plant-wide process, internal parameters of a principle model must be obtained and updated. The actual production process can infer a real model through idealization Approximation. Subsequently, a series of formulae are abstracted from this hypothetical model and constitute a principle model. The correlation between the principle model and actual collected data needs to be ensured by at least one optimal parameter. The optimization model is perfect by iterative optimization using improved PSO.

### Process control strategy

**Figure 5:**
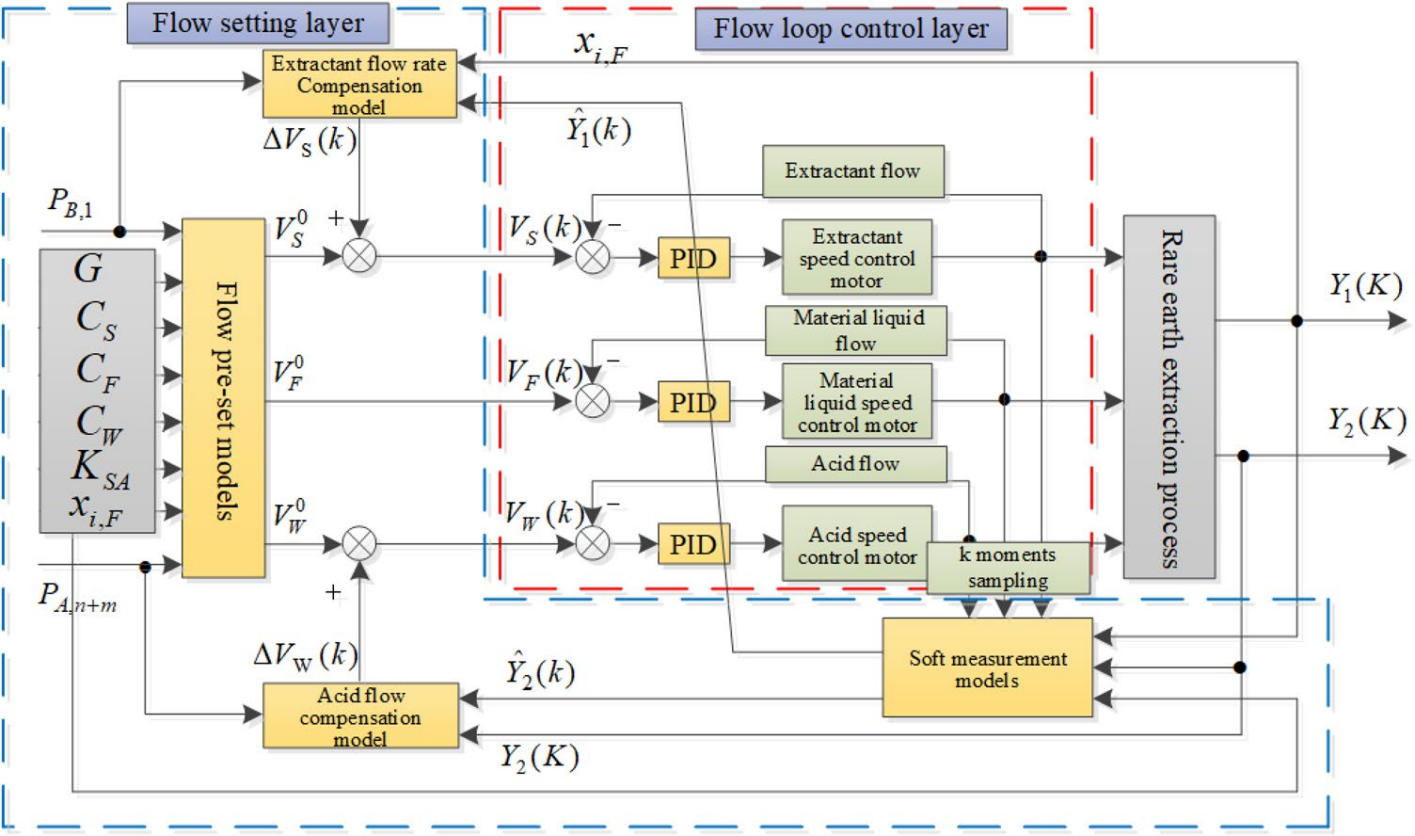
Control strategy for extraction process twin.

The current production process has a low level of automation and production optimization relying on manual experience. In order to guarantee the quality of the extracted products, it is necessary to design a dosing control strategy with optimization function. Yang proposed the component content distribution profile control which can be automatically regulated by dynamically compensating the related extract or scrubbing liquid flow-rate^36^. Lu proposed a model based on generalized prediction of the content of rare earth extracted components to meet the demand of component content floating in a specific interval^37^. A paper applied Static Setting and dynamic compensation based optimal control strategy for the Flow Rate of the Reagent in Ce Pr/Nd Extraction Process^38^. It can be depicted as Fig. 5. The control strategy in this thesis can quickly generate optimized control parameters based on real-time measured component content and historical cases, which is more practical than previous studies.

Staff usually rely on operational experience to pre-set the extractant and detergent flow rate values based on the raw material processing volume, rare earth feed liquid element distribution, saponification degree, extractant concentration, feed liquid concentration, acid concentration, and other entry conditions during the actual production process of the entire rare earth extraction. The control system obtains the detergent and acid settings from the case library that best match the real process based on the production parameters of the actual production process. CBR(Case-based reasoning) is an artificial intelligence reasoning strategy that uses human thinking to gather expert knowledge from instances in order to solve new issues. The case inference approach is utilized to predetermine the extractant/detergent for the rare earth extraction procedure based on this. Using case-based reasoning, the starting value of the extractant and detergent for the rare earth extraction method was derived. To decide which class a new instance belongs to, the classifier based on CBR approach is primarily focused on the known classes in of a known class. The data demonstrates that the first production parameters, Process Indicators, both have a substantial influence on the initial setup of extractant and acid by studying the process mechanism and process circumstances of the rare earth extraction manufacturing process. Each case consists of a description of the working conditions and a case solution stated by Eq. (23):

23 $$\begin{aligned} Case(X_{k},V_{k})=Case((x_{x_{1,x},\ldots ,x_{7,k}}),(v_{1,k},v_{2,k})) \end{aligned}$$

$$X_{k}=(x_{1,k},\ldots ,x_{7,k})$$ denotes the kth condition description. $$x_{1,k},\ldots ,x_{7,k}$$ presents all initial production parameters, Process Indicators. $$Y_{A}$$ refers the rare earth material distribution, $$C_{F}$$ refers raw material Liquid concentration,$$C_{S}$$ refers Extractant concentration, $$C_{W}$$ refers Detergent concentration, *G* refers Material and liquid handling capacity,$$P_{B1}$$ and $$P_{A,n+m}$$ both are Product purity indexes. $$ v_{k}=(v_{1,k},v_{2,k})$$ enotes the solution corresponding to the kth condition description; $$v_{1,k},v_{2,k}$$ respectively denote the extraction volume preset $$V_s$$ and the washing volume preset $$V_{w}$$ respectively. The case retrieval strategy can employ the nearest neighbor search strategy. case retrieval strategy can employ the nearest neighbor retrieval strategy, by calculating the distance between the target case and the source case. The smaller the distance, the higher the similarity between the two cases. Multiple search strategies are feasible such as KNN (K-nearest neighbor) search strategy. Intelligent compensation model for initial flow setting using a preset model based on case-based reasoning. Then a relatively reasonable preset value of extractant and detergent flow rate can be obtained. but the model does not take into account of the working conditions caused by real-time external disturbances. To ensure the requirements of the production process, the staff needs to periodically adjust the settings of the extractant and detergent according to the color of the solution.

**Figure 6:**
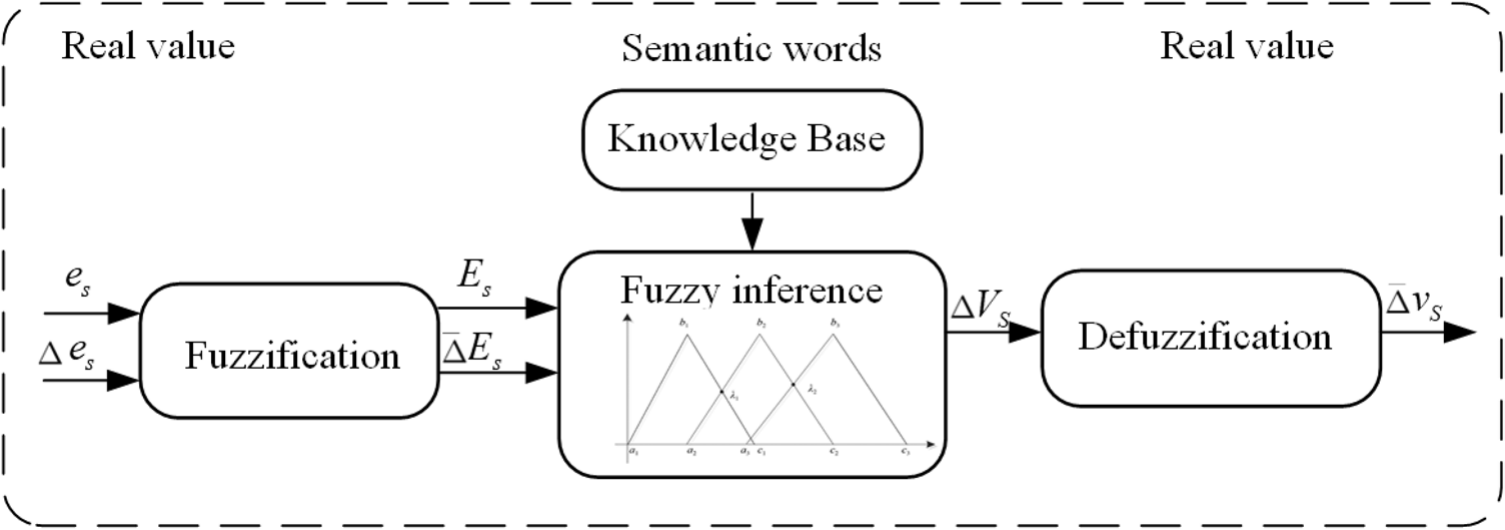
Compensated control based on fuzzy inferences.

Intelligent compensation model for initial flow setting is beneficial for the optimism of initial flow setting. The model mainly consists of a soft measurement model based on the color of rare earth solution and an intelligent compensation model based on fuzzy inference. The collected color images of rare earth solutions were extracted and converted to tree components. The H, S and I color components are extracted from the HSI color space. The color characteristics do not have a one-to-one correspondence with the component content, but the Least Squares Support Vector Machine can be applied in the Description of this correspondence since its excellent performance for modeling with small samples. To simulate the regulation of the initial flow by expert experience, a fuzzy control method can be utilized to build an intelligent compensation model. The process is presented as Fig. 6.

The component content $Y_{B}$ and $Y_{A}$ obtained from soft measurement model are compared with target component content $P_{B,1}$ and $P_{A,n+m}$. The error can presented as formulas $e_{s}=Y_{A}(K-1)-P_{A,n+m}$, $e_{w}=Y_{B}(K-1)-P_{B,1}$. The Variation of error show as $\Delta e_{s}=Y_{A}(K-1)-Y_{A}(K-2)$, $\Delta e_{s}=Y_{A}(K-1)-Y_{A}(K-2)$ The variation is set as the input of the fuzzy control model and the variation of extractant and acid is available as output.

## Model validation and case study

### Soft measurement model validation of solution component content

In this paper, the solutions in 100 extraction tanks are collected as experimental samples, and after collecting the solution images, light compensation is performed with soft measurement modeling.

**Figure 7:**
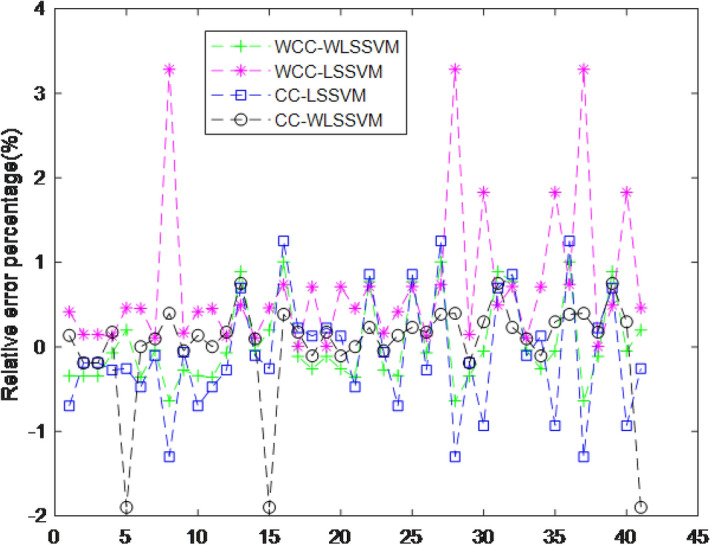
Relative error percentage of WLSSVM.

After extracting the HSI and RGB features of the compensated images, they are substituted into the WLSSVM model to obtain the predicted output of the component content. To Evaluate the accuracy of the soft-measurement model by comparing the actual assay values with the soft-measurement component content values. Relative error percentage as the Critical metrics for evaluating models as shown in Fig. 7. In order to verify the proposed method, the experiments are conducted in four cases, WCC means without color compensation, and CC means with color compensation. The experiments show that CC-WLSSVM has the lowest relative error compared to the actual value and the soft measurement effect is the last.

### Validation of process simulation methods

**Figure 8:**
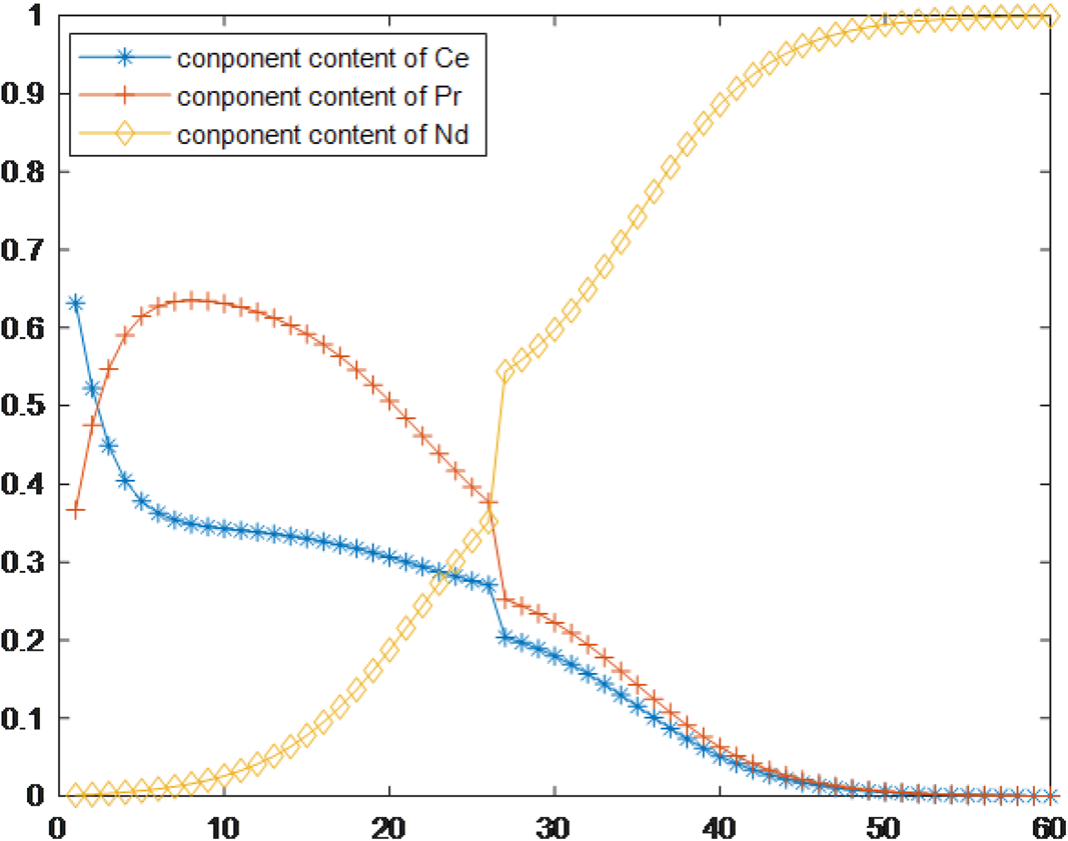
Predicted values of component content for each stage.

The process parameters set in this paper are as follows: The Ce component content of the inlet is 0.445,the Pr component content of inlet is 0.1842, and the Nd component content of inlet is 0.3713. The separation coefficient between Ce and Pr is 2.03 and The separation coefficient between Pr and Nd is 1.55. The number of extraction stages n is 26 and the number of washing stages m is 34.The extraction amount is 0.9995 and the export index is 0.9995. The parameters of the improved PSO are set as: acceleration constants c1 is 1.3,c2 is 1.7,wend is set as 0.86 wstart is set as 1,$\alpha$ is 0.7.The predictive values of the component content of rare earth components at each stage are shown in Fig. 8.

To further evaluate the prediction effect of the model, In this paper, we use the mean relative error and the maximum relative error to evaluate the deviation of the model from the actual value, and the root mean square error to reflect the degree of deviation as Eqs. (24)–(26). z is the model output value, z* is the actual component content. Relative error of each component after calibration as shown in Table 1.

24 $$\begin{aligned} MEANRE=   \frac{1}{n+m}\sum _{i=1}^{n+m}\frac{\left| z-z^{*} \right| }{z^{*}}\times 100 \end{aligned}$$

25 $$\begin{aligned} MAXRE=  MAX\left( \frac{\left| z-z^{*} \right| }{z^{*}} \times 100\% \right) \end{aligned}$$

26 $$\begin{aligned} RMSE=  \sqrt{\frac{1}{n+m}\sum _{i=1}^{n+m}\left( z-z^{*} \right) ^{2}} \end{aligned}$$

**Table 1:** Relative error of each component after calibration

Component content	MEANRE(%)	MAXRE(%)	RMSE(%)
Ce	0.425422	4.434351	$1.382\times 10^{-4}$
Pr	0.417555	3.656577	$2.246\times 10^{-4}$
Nd	0.175327	3.269205	$1.439\times 10^{-4}$

**Figure 9:**
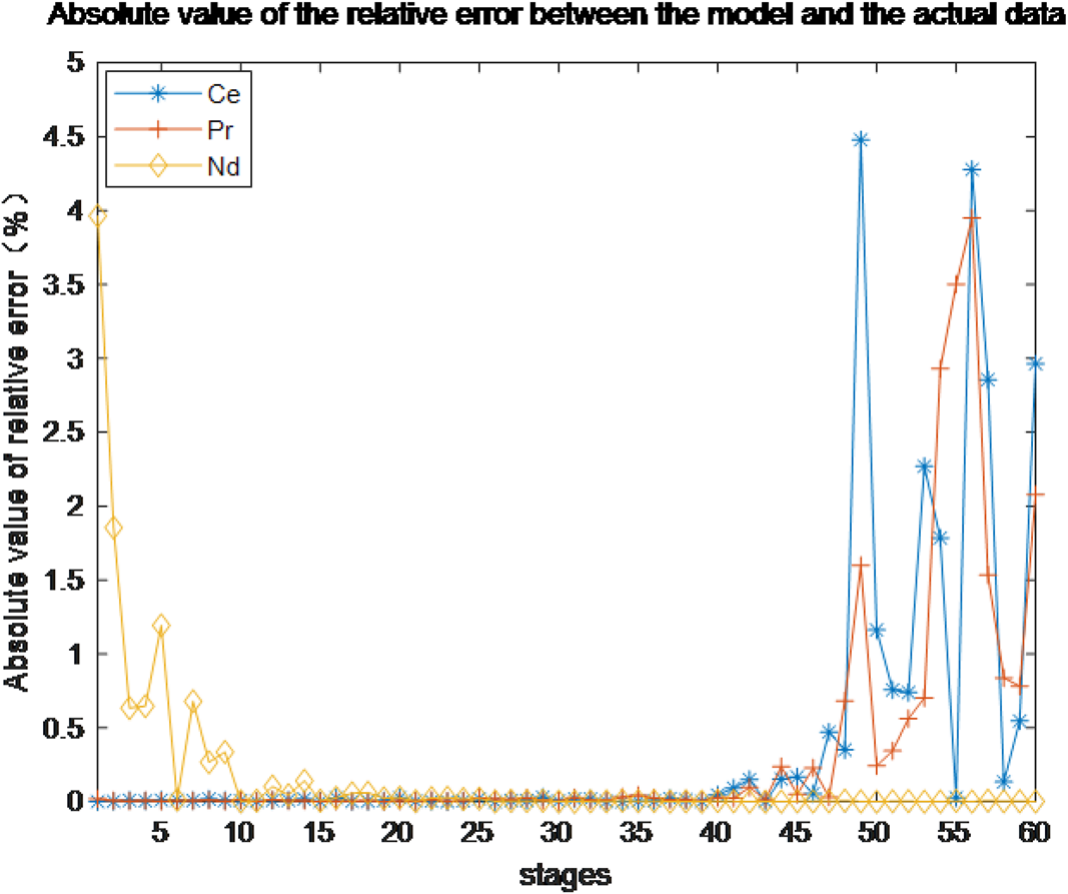
Absolute value of the relative error between the model and actual datal twin.

The absolute value of the relative error between the prediction results and the actual data is shown in Fig. 9. If the maximum relative error of the component content of each level does not exceed 5%, the error of the model is deemed to lie within a reasonable error range, and it can be seen from the figure that the error of the established model is within the normal range.

### System development and case study

**Figure 10:**
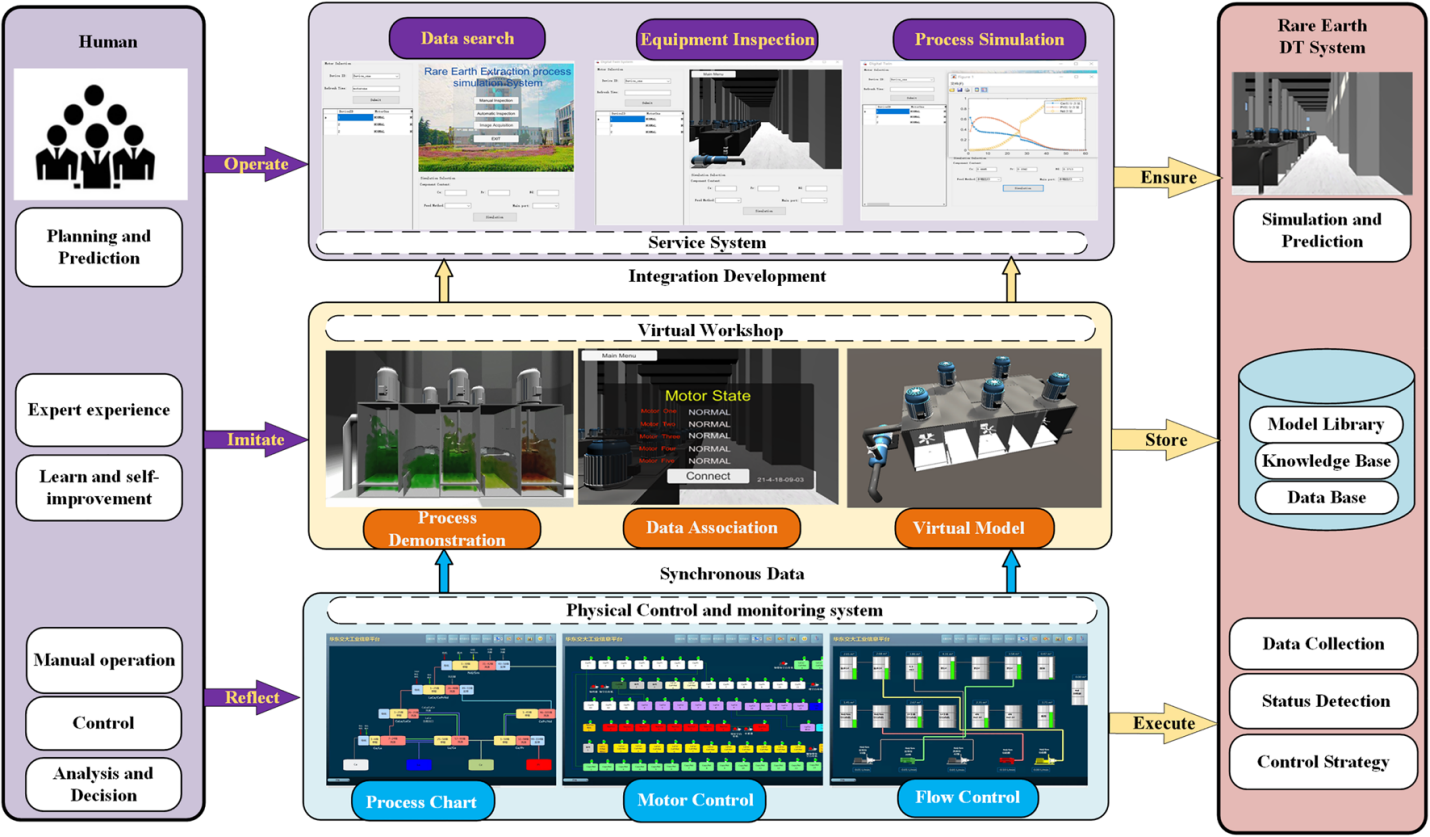
Test of rare earth DT system.

The specific applications include process simulation, virtual inspection, and data search. Finally, the contributions and limits of this research are examined.To further illustrate the efficacy of the aforementioned technique and framework, this study will perform experiments in the following areas: physical control and monitoring system, virtual workshop, service system, and human-DT connection. Figure 10 depicts the total framework diagram.

This paper is based on an existing information management system for rare earths production that has been enhanced to enable the sensing, control, and data gathering tasks of the digital twin plant. Configuration software such as WINCC can be used to implement the module. The Physical control and monitoring system is made up of three parts: the flow chart, the motor control module, and the flow control module. Here, the flow chart displays all procedures for monitoring each advice, such as the mixing motor and the extraction tank, and allows users to easily access the control and monitoring interface for the related process. The flow control and motor control modules generate reference control parameters such as material flow, extractant flow, and detergent flow. To develop control optimization strategies, these two modules can use the control optimization technique outlined above. The real-time data is synced to the database every two hours with the changeover of communication equipment for further analysis and utilization.

**Figure 11:**
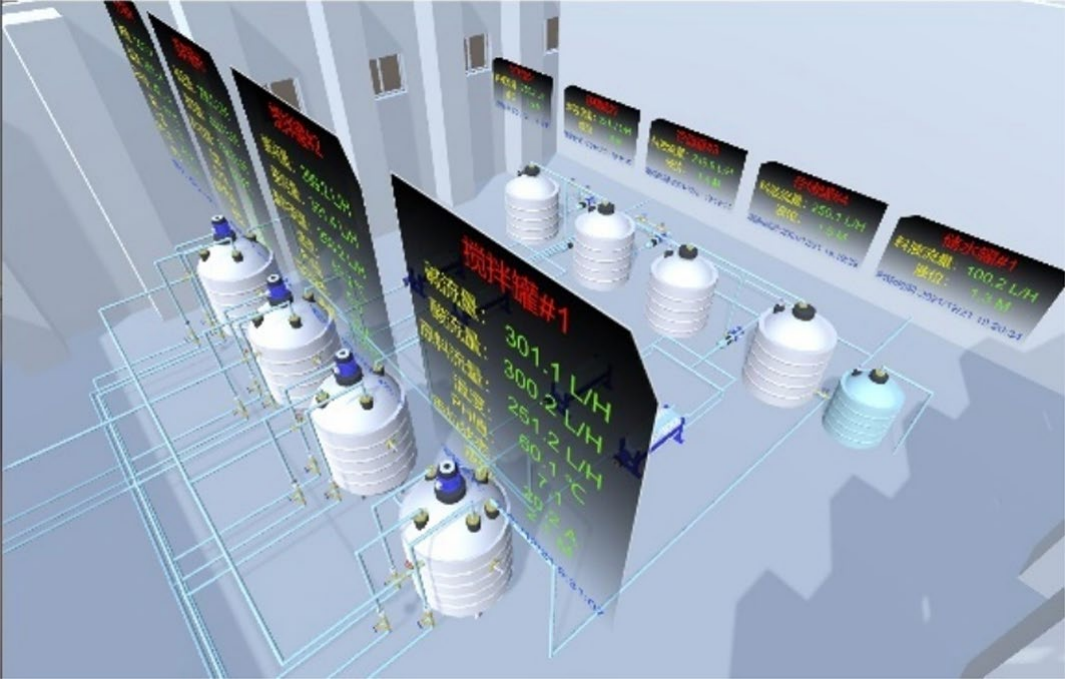
virtual workshop for rare earth process.

Virtual workshop is mainly convenient for users to quickly inspect the status of equipment in each workshop, and real-time working conditions which is integrated into the DT service system.The rare earth production line’s virtual workshop module is created by merging a 3D model with real-time data. Virtual-real interaction, data synchronization, and consistent behavior with Physical Equipment are the major goals of the virtual workshop . As a result, the virtual workshop may be broken down into three sections: process demonstration, data association, and virtual model. This section mostly relies on 3Dmaxs and Unity software to fulfill its goals. Demonstrating the change and distribution of materials liquid in the manufacturing process, which is accomplished by Fluid Animation in Unity, is a process demonstration. Data Association is realized by C# scripts about connection of data base with virtual model. The virtual system enables free inspection of each equipment and fast warning of abnormal working conditions, which greatly reduces production risks and unnecessary waste. To the end, A virtual workshop that allows data synchronization and free inspection is shown as Fig. 11.

The digital twin service system mainly realizes process simulation of rare earth production, virtual inspection, and soft measurement data retrieval of component content.The virtual workshop module, algorithms, and C# scripts, on the other hand, are enclosed inside subroutines for DT service system calls. A state searching application that can quickly display the states of motors retrieved from a database. To avoid possible failures in the process, the system may scan motor states using the time stamp and device ID and provide the predictive outcome of the component malfunction. The purpose of the virtual inspection is to guarantee that each piece of equipment receives a free and intuitive check, as well as a defect alarm. The process simulation is divided into two major subsystems: component content prediction for the extraction process and soft component content measurement. The proposed model with compensating coefficient is used to forecast component content.

Process simulation is the core function of DT system, to realize this function, it is necessary to solve the problem of soft measurement of component content. A subsystem was built based on the previously described soft measurement method for component content and Existing Soft measuring equipment and software.

**Figure 12:**
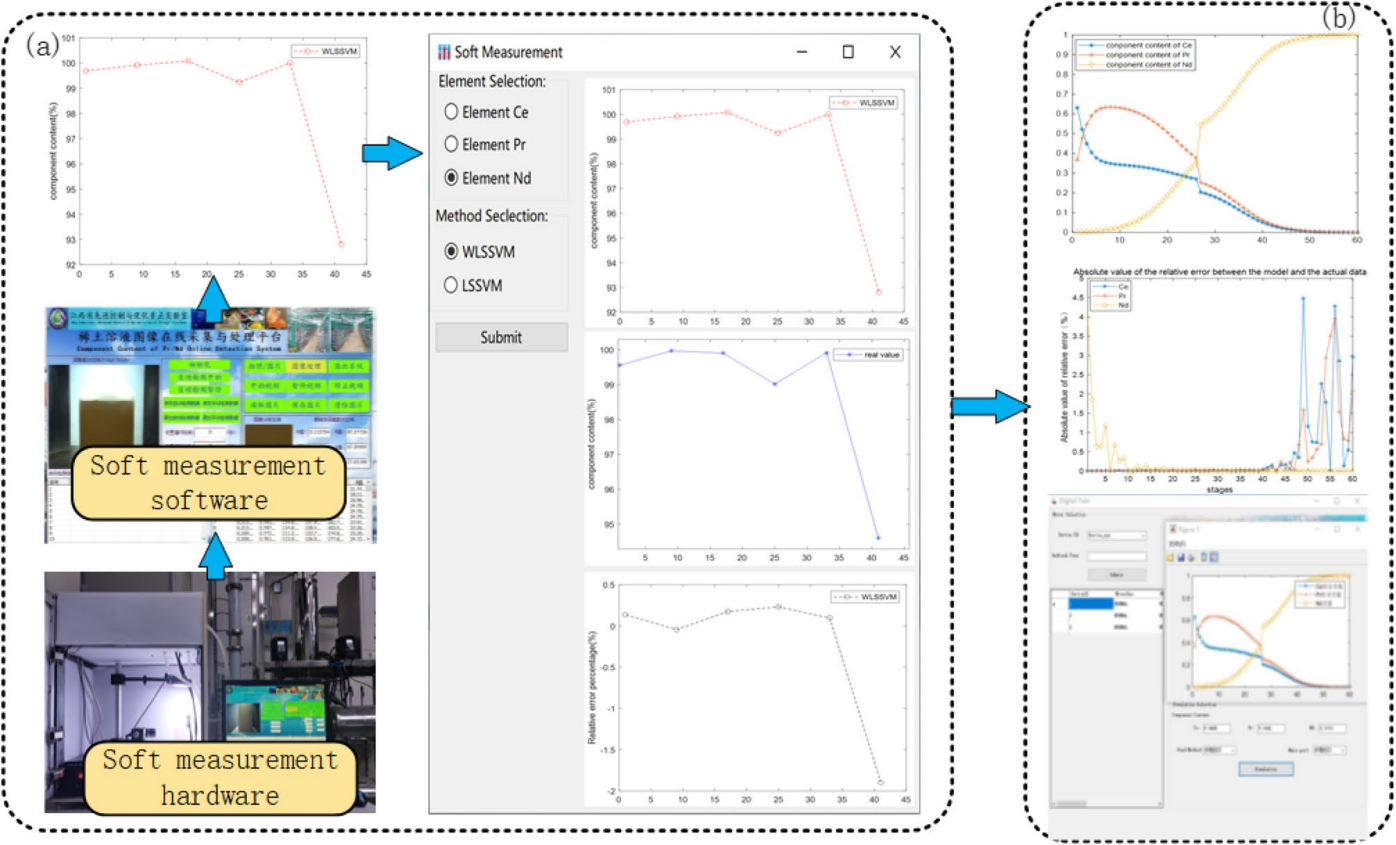
subsystem of DT service system (**a**) soft measurement of component content (**b**) process simulation of component content.

After extracting the HSI and RGB features of 6 compensated images collected from Detection level, those are substituted into the WLSSVM model to obtain the predicted output of the component content. To Evaluate the accuracy of the soft-measurement model by comparing the actual assay values with the soft-measurement component content values. The results are shown in the Fig. 12a.

From Relative error percentage of soft measurement,it can be seen that the model is able to accurately predict the component content based on the RGB and HSI characteristics of the solution graph.In this paper, 200 groups of component contents were detected as the training set of the process simulation model. A mathematical model with parameter optimization for component content prediction can be achieved using the above method. In order to verify the applicability of the model, some parameters are preset, a large amount of actual data is provided to train the model, and finally the prediction effect of the model is evaluated using the data from the validation set. The predictive values of the component content of rare earth components at each stage are shown in Fig.12b. It can be seen from the error of the established that model is within the normal range.

The developed rare earth digital twin service system has greatly reduced the inspection time and detection time, the inspection time from the original 30 min to 5 min, and the component content detection time from the original 1 h to 10 min.

## Conclusions

Rare earth production process relies heavily on manual labor for solution component content detection, process control and equipment inspection, and the production process has a large time lag and is prone to abnormal working conditions caused by untimely troubleshooting, which eventually affects the output quality of products. This paper proposes a soft measurement method of solution component based on the color characteristics of rare earth solution and a component process simulation method based on mechanism compensation for the problem of untimely detection of solution components, and adopts a case-based control optimization strategy for the problem of difficult control optimization, and builds a virtual scene to facilitate users to quickly understand the process and equipment conditions in order to reduce manual inspection. The soft measurement method of solution components is easily affected by the light and transparency of the container. The following points will be improved in future studies: Soft measurement method for solution fraction is slow to calculate and accuracy needs to be enhanced.The process simulation algorithm will be further improved to enhance the simulation accuracy. The control effect of the control strategy needs to be further improved, and the integration degree of the current DT system. The usability of the virtual workshop need to be enhanced. The interface will be presented in the form of a web page, and the server will complete the calculation and prediction.

## Data Availability

The datasets generated and analysed during the current study are not publicly available due to sensitive data involving production units but are available from the corresponding author on reasonable request.
